# Gastrointestinal Symptoms of Patients with Fabry Disease

**DOI:** 10.1155/2016/9712831

**Published:** 2015-12-31

**Authors:** Licia Pensabene, Simona Sestito, Angela Nicoletti, Francesca Graziano, Pietro Strisciuglio, Daniela Concolino

**Affiliations:** ^1^Department of Pediatrics, “Magna Graecia” University, Viale Pio X, 88100 Catanzaro, Italy; ^2^Translational Medical Sciences, Section of Pediatrics, “Federico II” University, Naples, Italy

## Abstract

In order to characterize gastrointestinal (GI) symptoms of 50 patients with Fabry disease (FD) (22 M; age range: 4–70 y; 35 adults and 15 children), validated questionnaires of GI symptoms were used to diagnose the functional gastrointestinal disorders (FGIDs) of the patients with GI symptoms (33/50 (66%); 25/35 adults and 8/15 children) according to Rome III criteria. In 16/25 of these adults and 2/8 of these children, the symptoms mimicked FGID. The adult subgroup included patients with unspecified functional bowel disorder (*n* = 9), functional bloating (*n* = 7), and IBS (*n* = 5), and the child subgroup included patients with abdominal migraine (*n* = 1) and IBS (*n* = 1). Among the 25 adults, 14 reported feeling full after a regular-size meal, and 12 complained of abdominal bloating/distension. All of the children with GI symptoms complained of low abdominal pain associated with changes in the form of the stool/improvements with defecation. In conclusion, according to Rome III criteria, the most frequent diagnoses of FGID among the adults with FD were unspecified functional bowel disorder, followed by functional bloating and IBS. The most frequent GI symptom in the children in our population was IBS-like abdominal pain, while the adults exhibited a full feeling following a regular-size meal and abdominal bloating/distension.

## 1. Introduction

Fabry disease (OMIM 301500) is a progressive, X-linked inherited disorder of glycosphingolipid metabolism that results from deficient or absent lysosomal *α*-galactosidase A activity due to progressive accumulation of globotriaosylceramide (Gb3) and related glycosphingolipids (galabiosylceramide) in nearly all organ systems. The incidence of this disease has been reported to be between 1 in 40,000 and 1 in 120,000 [1]; however, recent newborn screening surveys suggest that the incidence may be as high as 1 : 3,100 in male newborns [2]. In the past, most “carrier” females have typically been thought to be clinically unaffected, but it is now well proven that females with FD are at a significant risk for major organ involvement and decreased quality of life [3]. Lysosomal accumulation of Gb3 begins in utero [4], and signs and symptoms of FD emerge in childhood and adolescence. The earliest presenting symptoms are typically neuropathic pain, primarily burning paroxysmal hand and foot pains (acroparesthesias), and gastrointestinal (GI) problems, which can substantially influence health-related quality of life [5, 6]. Disorders of sweat secretion [7, 8] leading to impaired thermoregulation involving heat and exercise intolerance, corneal opacities, and microalbuminuria have also been observed. Several years later, typical skin lesions (angiokeratoma) appear, and progressive damage to the vital organ systems develops with age and leads to organ failure. End-stage renal disease and life-threatening cardiovascular or cerebrovascular complications limit life expectancy [9–11]. A wide range of phenotypic variabilities in terms of both target-organs and the severity of FD has been reported even between family members harbouring the same mutation [12]. Enzyme replacement therapy (ERT) has been available since 2001 and has been validated as an effective therapeutic approach that is able to stabilize organ function [13, 14], reduce the severity of symptoms [15], and improve the quality of life [16]. A scoring system has been proposed to evaluate improvements in patients [17]. Moreover, it has been suggested that initiation of ERT in childhood might slow or stop the progression of organ damage before irreversible changes occur [5]. Therefore, the early diagnosis of FD is extremely important to enable the earlier onset of ERT [6]. However, because the presenting signs and symptoms of FD are described as nonspecific (e.g., acroparesthesias and nonspecific GI symptoms), the diagnosis may be delayed, particularly in the absence of a family history of FD. Approximately 60% of patients who manifest FD report GI symptoms [10], and GI symptoms are often among the earliest presenting symptoms of FD [18–23]. Gastrointestinal symptoms in the course of FD have also been reported to obscure other characteristic symptoms [24]. The most commonly reported GI symptom in affected patients is diarrhoea with frequent loose bowel movements and cramping abdominal pain. In many patients, episodes of diarrhoea are interspersed with periods of normal, or even reduced, bowel activity during which they may complain of constipation, which results in an alternating pattern associated with abdominal pain that is reminiscent of irritable bowel syndrome (IBS). Patients may also experience a sense of early satiety after meals, epigastric discomfort, and abdominal bloating. Again, the symptoms of abdominal discomfort and bloating associated with meals are features of IBS [25], which is a functional GI disorder (FGID). FGIDs are conditions that include variable combinations of chronic or recurrent GI symptoms that are not explainable by structural or biochemical abnormalities [26]. In 2006, the new and validated questionnaires on pediatric GI symptoms and adult GI symptoms, both of which are based on Rome III Criteria [27], were published to enable the correct diagnoses of FGIDs in these two age subgroups.

The aims of this study were to characterize the GI symptoms of paediatric and adult patients with FD using specific tools, such as the questionnaires on GI symptoms based on Rome III criteria, and to identify a specific pattern of GI symptoms that is useful for the earlier diagnosis of FD.

## 2. Methods

After providing informed consent, 50 patients with FD were recruited for this study. Twenty-three of the 30 FD patients followed up at our institution (the Department of Pediatrics of the University Magna Graecia of Catanzaro) who were informed during outpatient clinical visits and 27 of 105 members of the patients of Anderson-Fabry Italian Association (AIAF) who were informed during the annual meeting of the association agreed to participate in this study and completed the provided questionnaires [27].

Among the fifty enrolled FD patients, 22 were male, the age range was 4–70 y, 15 were children (five males, mean age: 11.8 y, range 4–18 y), and 35 were adults. Informed consent was obtained from all adult patients and from the parents of the children of any age. In all patients, the diagnosis of Fabry disease was made via measurements of leukocytes and/or plasmatic *α*-galactosidase A activities and molecular analysis of the *α*-galactosidase A gene. Eighty per cent of the patients exhibited classical genotypes (40/50), and the other 20% exhibited late onset disease (10/50) (Table 1).

After receiving informed consent, two validated questionnaires [27] (one about paediatric GI symptom and the other about adult GI symptoms) were used to detect GI symptoms mimicking FGIDs according to Rome III criteria among the 33 FD patients with GI symptoms (eight children with a mean age of 12.25 years and 25 adults). The questionnaire on pediatric GI symptoms-Rome III version (QPGS-RIII) is an adapted and abbreviated version of the questionnaire on pediatric gastrointestinal symptoms (QPGS) [28, 29] that was developed with the support of a grant from the Rome Foundation and that has undergone preliminary validation [30, 31]. The QPGS-RIII is a validated, age-appropriate, structured questionnaire that includes sections assessing children's bowel habits, abdominal pain, and other gastrointestinal symptoms as well as limitations in activities using 5-point scales to measure the frequency, severity, and duration of symptoms. The parent-report version of the QPGS-RIII was completed by the parents of children between 4 and 10 years of age, and the self-report version of the QPGS-RIII was completed by FD children 10 years of age and older. The questionnaires were scored using the age-appropriate scoring algorithms [27] to assess whether each patient met the criteria for each individual FGID. Similarly, the questionnaire on adult GI symptoms-Rome III version is a validated adaptation of the original questionnaire that was developed according to Rome II criteria and is therefore a valid and reliable instrument for provisional diagnoses of all FGIDs [27]. The questionnaire on adult GI symptoms-Rome III version was completed by the adults with FD and GI symptoms and was scored using the age-appropriate scoring algorithms [27] to assess whether each patient met the criteria for each of the individual FGIDs. In all cases, even when a diagnosis of FGID was not possible according to Rome III criteria, the patterns of GI manifestations were evaluated for all paediatric and adult patients and were analysed separately for the two age subgroups.

For those patients receiving enzyme replacement therapy (ERT), careful histories were taken to evaluate any possible changes in GI symptoms after the initiation of ERT.

## 3. Results

**Figure 1:**
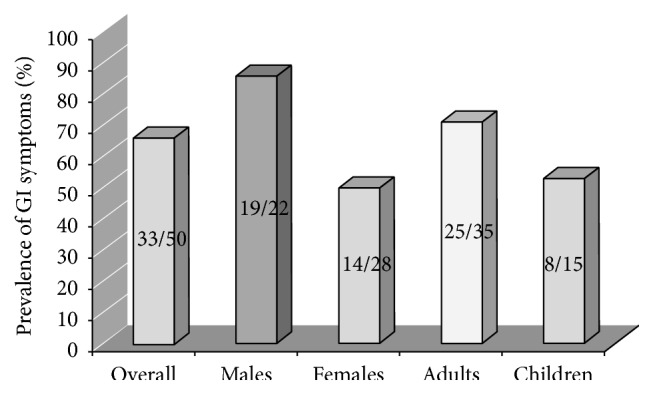
Prevalence of GI symptoms among 50 patients with FD.

The phenotypes, genotypes, enzyme activities, and clinical details of all patients enrolled in our study are reported in Table 1. The overall prevalence of gastrointestinal complaints in our FD patients is illustrated in Figure 1. No differences in GI symptoms were identified between the patients with the classic phenotype and those with milder mutations. The onset of GI symptoms in our patients occurred at a median age of 10 years. At the time of enrolment in the study, 28/33 of the patients with FD and GI symptoms (four children and 24 adults) had been on ERT for a mean of 4.8 years (range: 2–8 years).

**Figure 2:**
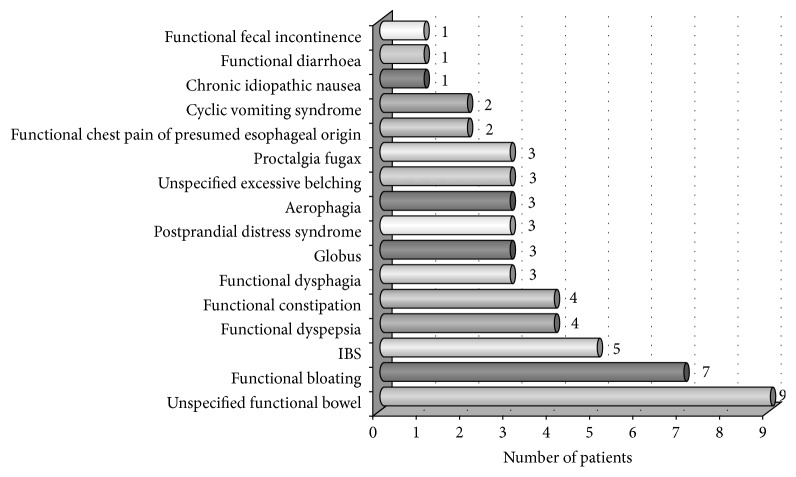
Prevalence of GI symptoms mimicking each subtype of FGID in the adult patients with FD.

Among the 33 FD patients with GI complaints, 16/25 adults and 2/8 children presented GI symptoms that mimicked FGIDs according to Rome III criteria. The prevalence of GI symptoms that mimicked each subtype of FGID in the adult patients with FD is shown in Figure 2. Of the two children with GI symptoms that mimicked a FGID, one was diagnosed with abdominal migraine, and the other was diagnosed with IBS.

The patterns of GI manifestations of all paediatric and adult patients were evaluated based on the answers to the questionnaire and were analysed separately in the two age subgroups.

Specifically, in the adult subgroup, the symptoms that were reported to have persisted for at least the last 3 months were the following:

- Fourteen patients complained of feeling uncomfortably full after a regular-sized meal. Eight of these 14 patients had experienced this feeling for 6 months or longer, and seven had been unable to finish a regular-sized meal in the last 3 months.
- Thirteen patients complained of having less than three bowel movements per week (11 experienced this symptom only occasionally), and 14 reported hard or lumpy stools (11 only occasionally).
- Twelve patients reported bloating or distension (8/12 at least 2-3 days/month) for 6 months or longer.
- Twelve patients reported pain or discomfort anywhere in their abdomen. Nine of these 12 patients experienced these symptoms at least one day per month, these symptoms limited the daily activities of four patients, and 7/12 patients had experienced these symptoms for 6 months or longer. In 10 patients, this discomfort or pain improved or disappeared following a bowel movement, 8/12 patients reported more frequent bowel movements upon the onset of this discomfort or pain, 7/12 patients reported looser stools, and 2/12 reported harder stools.

In the child subgroup, all of the eight patients with FD and GI symptoms had experienced a belly ache or pain in the area around or below the belly button within the last 2 months. Specifically five of these patients complained of these symptoms 1 to 3 times per month, two complained of these symptoms once per week, and one complained of these symptoms every day. Four children reported that these symptoms had persisted for 1 year or longer, two children reported experiencing symptoms for 4 to 11 months, one child reported a duration of 3 months, and one child reported experiencing symptoms for 1 month or less. Six children reported that within the last 2 months the belly ache improved following a bowel movement. Three of these children reported that this improvement always occurred, two reported that it occurred most of the time, and one reported that it occurred only occasionally. Five of the eight children had softer and mushy or watery stools when they had these symptoms, and three of these children reported occasionally having more stools than usual; the other two reported having fewer stools than usual most of the time. For one child, within the last 2 months, his stools were harder or lumpier than usual occasionally when he had these symptoms, and he occasionally had fewer stools than usual.

## 4. Discussion

FD is a rare multisystemic inherited metabolic disease with profound effects on almost all organ systems, reduced life expectancy [32], and substantial involvement of the gastrointestinal tract [19, 23]. The pathophysiology of the altered GI function in FD is poorly understood, although it is plausible that the accumulation of Gb3 in the neurones of the submucosal and myenteric nerve plexuses causes enteric neuropathy. Gb3 also accumulates in the intestinal smooth muscle, and a direct myopathic effect or a combined myopathy and neuropathy may be important [33]. Enteropathy affecting the sympathetic and parasympathetic divisions of the autonomic nervous system is also possible, and autonomic nerve involvement has been reported in FD [34]. The prevalence of GI symptoms in our patients (66%) was higher than what has previously been described; GI symptoms have been reported in approximately 60% of hemizygous males [34, 35] and between 30% and 60% of heterozygous females [34]. A potential explanation for this discrepancy is that the reporting of symptoms in our groups of patients was elicited by direct specific questions that were asked to characterize GI manifestations. Most of the available data on this topic were retrospectively collected from one of the available databases on Fabry disease. Because these databases were primarily developed for the follow-up of patients on ERT, they often lack precise information about specific organ complaints, such as those related to the gastrointestinal system. Hence, information obtained using specifically developed questionnaires may be helpful to better understand the symptomatology. The predominant previously reported GI symptoms in FD are diarrhoea and abdominal pain, which mimic the functional gastrointestinal disorder diarrhoea-predominant IBS [36]. Of course, patients with FD have an organic underlying disease that can explain the clinical picture and could be considered an exclusion criterion for the diagnosis of an FGID; however, our aims were to characterize the GI symptoms of patients with FD using the questionnaires on GI symptoms according to the Rome III criteria and to evaluate whether this process could eventually identify a pattern of GI symptoms that can be used as a diagnostic tool to select patients who are more likely to have FD and thus allow for the earlier diagnosis of FD. According to Rome III criteria, 64% of our adults and 25% of our children with FD presented GI symptoms that mimicked FGIDs. In our adult group of patients with FD, the most frequent FGID diagnosis was unspecified functional bowel disorder, followed by functional bloating and IBS. The diagnosis of unspecified functional bowel disorder includes bowel symptoms not explained by an organic aetiology that do not meet Rome III criteria for the other defined categories. Moreover, functional bloating, that is, the second most frequent FGID diagnosed in our adult patients, is characterized by a recurrent feeling of bloating or visible distension for at least 3 days/month within 3 months and the absence of the criteria for diagnoses of functional dyspepsia, irritable bowel syndrome, or functional constipation. Neither of these subtypes of FGID has a specific clinical picture or requires the meeting of Rome III criteria for other defined categories (except for the presence of bloating in functional bloating disorder). The third most frequent FGID in our adult patients was IBS, the prevalence of which was markedly higher than what has previously been described in the general population. Surveys of adult European and North American populations have estimated the prevalence of IBS to be in the range of 7% to 22% of adults [27, 37]; therefore, our results suggest that GI manifestations in FD adult patients may frequently mimic this subtype of FGID. Instead, in our children with FD, the GI symptoms mimicked specific FGIDs, such IBS and abdominal migraine, in two of the eight patients. Among the GI manifestations in our adults with FD, the most frequent GI symptom was a “full feeling after a regular-size meal,” followed by “constipation-like symptoms” (fewer than three defecations/week and/or hard or lumpy stools; however, the majority of these patients reported that these symptoms occurred only occasionally) and bloating or distension. Therefore, in our adult patients, abdominal pain and diarrhoea were not the most frequent symptoms as has previously been described [23]. Instead, all of our eight children with GI symptoms complained of belly aches or pains in the area around or below the belly button, and these symptoms improved following bowel movements in six of these children. Therefore, we consider this clinical picture to be an IBS-like abdominal pain, even if only one child fulfilled Rome III criteria for IBS. Finally, paediatricians and internists commonly misdiagnose Fabry disease, and for this reason, recurrent abdominal pain in childhood or adolescence should include Fabry disease in the differential diagnosis to facilitate the earlier diagnosis and treatment of these patients.

In conclusion, the systematic application of the validated questionnaires on GI symptoms in patients with gastrointestinal complaints can be used to better characterize these symptoms and to identify a subgroup of patients in which the presence of other signs or symptoms characteristic of FD should be determined. Specifically, when Rome III criteria do not enable the detection of a specific FGID (and gastrointestinal manifestations are likely due to an underlying disease) and even in adults with diagnoses of unspecified functional bowel disorder, functional bloating, or IBS and those who experience a full feeling after a regular-sized meal and in children with IBS-like abdominal pain, the presence of other signs or symptoms characteristic of FD (such as acroparesthesias, heat and exercise intolerance, corneal opacities, microalbuminuria, and angiokeratomas) should be carefully examined. In such cases, determination of *α*-gal activity must be considered among the additional tests required for diagnoses of other GI organic diseases. This approach could be useful for ensuring earlier diagnoses of FD before irreversible organ damage is present and can lead even inexperienced doctors to diagnose this rare metabolic disease in the early stages to initiate early ERT.

## Figures and Tables

**Table 1:** Phenotype, genotype, enzyme activity, and clinical details of 50 patients with Fabry disease.

Patient	Age	Sex	Plasmatic enzyme activity (nmol/mL/h) (in males)	Genotype	Phenotype	GI symptoms
1	51	M	0,3	A288D	Classic	Yes
2	47	F		c.846_847 delTC	Classic	Yes
3	59	F		c.846_847 delTC	Classic	Yes
4	25	F		c.846_847 delTC	Classic	Yes
5	21	F		c.846_847 delTC	Classic	Yes
6	37	M	0,48	c.846_847 delTC	Classic	Yes
7	27	F		C172Y	Classic	Yes
8	31	M	3,7	C172Y	Classic	Yes
9	34	M	2,8	C172Y	Classic	Yes
10	28	M	3,4	C172Y	Classic	Yes
11	4	M	2,6	C172Y	Classic	No
12	60	F		C172Y	Classic	Yes
13	39	M	5,3	C172Y	Classic	Yes
14	47	M	0,9	D165H	Classic	Yes
15	14	F		D165H	Classic	No
16	22	M	0,4	Del 2b (#124-125)	Classic	No
17	39	M	1,77	E59K	Classic	No
18	12	F		E59K	Classic	No
19	16	F		G395A	Classic	No
20	14	F		G395A	Classic	No
21	45	M	1,5	G395A	Classic	Yes
22	8	F		G395A	Classic	Yes
23	16	F		IVS 4-1 (g->a)	Classic	Yes
24	40	M	0,8	IVS 4-1 (g->a)	Classic	Yes
25	10	M	0,7	IVS 4-1 (g->a)	Classic	Yes
26	18	F		IVS 4-1 (g->a)	Classic	Yes
27	42	M	0,7	IVS 4-1 (g->a)	Classic	No
28	19	F		M 51 I	Late onset	No
29	25	M	0,78	M 51 I	Late onset	No
30	53	F		M 51 I	Late onset	No
31	50	F		M 51 I	Late onset	No
32	28	F		M42V	Classic	Yes
33	20	F		M 51 I	Late onset	Yes
34	37	F		N215S	Mild, cardiac variant	Yes
35	10	F		N215S	Mild, cardiac variant	No
36	37	F		P409S	Classic	No
37	40	M	0,9	P409S	Classic	Yes
38	12	M	1,1	P409S	Classic	Yes
39	34	F		R112H	Late onset	Yes
40	9	M	0,8	R112H	Late onset	No
41	14	F		R112H	Late onset	Yes
42	30	M	0,1	R342Q	Classic	Yes
43	69	F		S78X	Classic	Yes
44	70	F		S78X	Classic	No
45	26	F		S78X	Classic	Yes
46	12	F		S78X	Classic	Yes
47	51	F		S78X	Classic	No
48	46	M	2,9	W162X	Classic	Yes
49	48	M	3,1	W162X	Classic	Yes
50	8	M	2,3	W162X	Classic	Yes
